# Comparative effects of flow vs. volume-controlled one-lung ventilation on gas exchange and respiratory system mechanics in pigs

**DOI:** 10.1186/s40635-020-00308-0

**Published:** 2020-12-18

**Authors:** Jakob Wittenstein, Martin Scharffenberg, Xi Ran, Diana Keller, Pia Michler, Sebastian Tauer, Raphael Theilen, Thomas Kiss, Thomas Bluth, Thea Koch, Marcelo Gama de Abreu, Robert Huhle

**Affiliations:** Pulmonary Engineering Group, Dept. of Anaesthesiology and Intensive Care Medicine, University Hospital Carl Gustav Carus at Technische Universität Dresden, Fetscherstrasse 74, 01307 Dresden, Germany

**Keywords:** OLV, FCV, VILI, Hypoxemia, Thoracic surgery, Intravascular hypovolaemia, Normovolaemia, Mechanical power, Resistive mechanical power

## Abstract

**Background:**

Flow-controlled ventilation (FCV) allows expiratory flow control, reducing the collapse of the airways during expiration. The performance of FCV during one-lung ventilation (OLV) under intravascular normo- and hypovolaemia is currently unknown. In this explorative study, we hypothesised that OLV with FCV improves PaO_2_ and reduces mechanical power compared to volume-controlled ventilation (VCV). Sixteen juvenile pigs were randomly assigned to one of two groups: (1) intravascular normovolaemia (*n* = 8) and (2) intravascular hypovolaemia (*n* = 8). To mimic inflammation due to major thoracic surgery, a thoracotomy was performed, and 0.5 μg/kg/h lipopolysaccharides from *Escherichia coli* continuously administered intravenously. Animals were randomly assigned to OLV with one of two sequences (60 min per mode): (1) VCV–FCV or (2) FCV–VCV. Variables of gas exchange, haemodynamics and respiratory signals were collected 20, 40 and 60 min after initiation of OLV with each mechanical ventilation mode. The distribution of ventilation was determined using electrical impedance tomography (EIT).

**Results:**

Oxygenation did not differ significantly between modes (*P* = 0.881). In the normovolaemia group, the corrected expired minute volume (*P* = 0.022) and positive end-expiratory pressure (PEEP) were lower during FCV than VCV. The minute volume (*P* ≤ 0.001), respiratory rate (*P* ≤ 0.001), total PEEP (*P* ≤ 0.001), resistance of the respiratory system (*P* ≤ 0.001), mechanical power (*P* ≤ 0.001) and resistive mechanical power (*P* ≤ 0.001) were lower during FCV than VCV irrespective of the volaemia status. The distribution of ventilation did not differ between both ventilation modes (*P* = 0.103).

**Conclusions:**

In a model of OLV in normo- and hypovolemic pigs, mechanical power was lower during FCV compared to VCV, without significant differences in oxygenation. Furthermore, the efficacy of ventilation was higher during FCV compared to VCV during normovolaemia.

## Background

During one-lung ventilation (OLV) the incidence of hypoxemia can be as high as 10% and may lead to adverse outcomes [1]. Hypoxemia results mainly from the formation of atelectasis in the dependent lung, which increases the intrapulmonary shunt and alveolar dead space [2]. Atelectasis can lead also to cyclic collapse and reopening of lung areas, the so-called atelectrauma [3]. Furthermore, in the presence of atelectasis, the tidal volume is distributed across a reduced lung volume, possibly resulting in hyper-distension of lung zones, or volutrauma [4]. Atelectrauma and volutrauma can increase the transfer of energy per time from the ventilator to the lungs (mechanical power [5]), which might result in ventilator-induced lung injury (VILI) [6]. In fact, the risk of VILI is increased during thoracic procedures compared with non-thoracic procedures [7].

Volume-controlled mechanical ventilation (VCV) is frequently used during surgery [8], but this mode does not limit the airflow during expiration. Un-controlled expiratory flow combined with avalanche-like lung closure dynamics [9] favours the formation of atelectasis. Flow-controlled ventilation (FCV) is a new mode of mechanical ventilation where the flow is controlled during both inspiration and expiration, and phases of zero flow are avoided. During two-lung ventilation in pigs, FCV increased oxygenation in non-injured [10] and injured lungs [11], but the performance of FCV during OLV has not been investigated. Theoretically, FCV would also reduce the mechanical power [12], but, to our knowledge, this effect has not been demonstrated.

In the present study, we aimed at determining the effects of FCV and VCV on gas exchange and respiratory mechanics during OLV in pigs. Since hypovolaemia may blunt the hypoxic pulmonary vasoconstriction [13], the performance of these mechanical ventilation modes was investigated during normovolaemia and hypovolaemia. In this explorative study, we hypothesised that FCV improves oxygenation, and reduces the mechanical power, as compared to VCV during OLV.

## Methods

The Institutional Animal Care and Welfare Committee and the Government of the State of Saxony, Germany (DD24.1-5131/449/71), approved the study, which was performed as the secondary protocol of another study on pulmonary perfusion. All animals received humane care in compliance with the Principles of Laboratory Animal Care formulated by the National Society for Medical Research and the US National Academy of Sciences Guide for the Care and Use of Laboratory Animals and complied with relevant aspects of the ARRIVE guidelines. Animals were kept at controlled temperature and light-dark cycle with free access to water and food.

### Experimental protocol

**Fig. 1:**
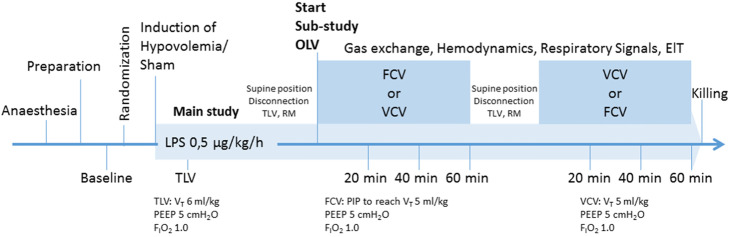
Time course of interventions and measurements. FCV, flow-controlled ventilation; VCV, volume-controlled ventilation; TLV, two-lung ventilation; LPS, lipopolysaccharide; EIT, electrical impedance tomography, PIP, peak inspiratory pressure. *V*_*T*_ = tidal volume; PEEP = positive end-expiratory pressure, F_I_O_2_ = fraction of inspired oxygen

The time course of the experiments is presented in Fig. 1. Sixteen female pigs (German landrace, weighing 35 to 49 kg, Danish Specific Pathogen Free Certification, www.spf.dk) were intramuscularly pre-medicated with midazolam (1 mg/kg) and ketamine (10 mg/kg). Intravenous anaesthesia was induced and maintained with midazolam (bolus 0.5 to 1 mg/kg, followed by 1 mg/kg/h) and ketamine (bolus 3 to 4 mg/kg, followed by 15 mg/kg/h). Muscle paralysis was achieved with atracurium (bolus 3 to 4 mg/kg, followed by 3 mg/kg/h). Lungs were initially ventilated in volume-controlled mode using the following settings: fraction of inspired oxygen (F_I_O_2_) of 1.0, tidal volume (V_T_) of 6 mL/kg, positive end-expiratory pressure (PEEP) of 5 cmH_2_0, inspiratory: expiratory (I:E) ratio of 1:1, constant gas flow of 25 L/min, and respiratory rate (RR) adjusted to achieve PaCO_2_ = 35–45 mmHg.

All skin incisions were preceded by infiltration of 2–5 mL lidocaine 2%. After surgical preparation of the right internal carotid artery, a PICCO catheter (20 cm; Pulsion Medical Systems SE, Feldkirchen, Germany) was inserted and the mean arterial pressure (MAP) continuously monitored. A 7.5 Fr. pulmonary artery catheter (Opticath, Abbott, Abbott Park, USA) to measure cardiac output (CO), and mean pulmonary artery pressure (MPAP) was advanced through an 8.5 Fr. sheath, placed in the right external jugular vein until typical pulmonary arterial pressure waveforms were observed. Urine was collected with a bladder catheter inserted through a median mini-laparotomy.

In order to measure regional transpulmonary pressures, three custom-made pressure sensors were attached to the parietal pleura in the following regions of the left hemithorax: (1) ventral (4–5th rib parasternal), (2) dorsal (4–5th rib paravertebral), and (3) caudal (8–9th rib paravertebral) as described in detail before (Kiss et al., 2019). Briefly, animals were positioned in the right lateral decubitus position. The lungs were separated introducing a left-sided double-lumen tube (39 Fr., Silbroncho Fuji, Tokyo, Japan) through a tracheotomy, with the bronchial tip placed into the left main bronchus. Pressure sensors were placed using video-assisted thoracoscopy (Thoracoscopy Set, Karl Storz, Tuttlingen, Germany). A thoracic drain was placed and connected to a surge tank.

To mimic thoracic surgery conditions a right-sided thoracotomy was performed between the medial-clavicular and the anterior axillary line in the 4–5th intercostal space and a rib spreader placed. Furthermore, systemic inflammation was induced by continuous administration of 0.5 μg/kg/h purified lipopolysaccharides from *Escherichia coli* O111:B4 (SIGMA Aldrich, St. Louis, USA) through the central venous line.

The intravascular volume was initially maintained with a crystalloid solution (E153, Serumwerk Bernburg AG, Bernburg, Germany) at a rate of 5 mL/kg/h. Norepinephrine was used to maintain a mean arterial pressure of at least 60 mmHg throughout the experiments.

Animals were then randomised to either the normo- or the hypovolaemia group. For induction of hypovolaemia, 25 % of the calculated blood volume, estimated as 70 mL/kg [14], was drawn from the central venous catheter. Thereafter, randomisation was performed and animals were placed in the left lateral decubitus position and submitted to one of two sequences, namely FCV-VCV or VCV-FCV (60 min per mode, crossover). To reset lung history, animals were put on supine position, disconnected from the ventilator, two-lung ventilation resumed, and an alveolar recruitment manoeuvre performed before the start of each ventilation mode.

### One lung ventilation

For VCV V_T_ of 5 mL/kg; F_I_O_2_ of 1.0, PEEP of 5 cmH_2_O, I:E of 1:1, a RR of 30-35 min^-1^ to achieve pH > 7.25 and flow of 25 L/min were chosen. FCV was performed with the Evone Ventilator (Ventinova Medical, Eindhoven, Netherlands) using a Tritube (Ventinova Medical), placed in the bronchial lumen of the double-lumen tube with the tip of the Tritube just before the end of the double-lumen tube. Peak inspiratory pressure (PIP) was set to achieve a V_T_ of 5 mL/kg. Furthermore, PEEP of 5 cmH_2_O, F_I_O_2_ of 1.0, I:E of 1:1, and flow were chosen to reach a RR of 30–35 min^-1^ to achieve pH > 7.25.

### Measurements

Following premedication and preparation (baseline) measurements of gas exchange, respiratory signals, haemodynamics and distribution of ventilation by electrical impedance tomography (EIT) were collected. Twenty, 40 and 60 min (20′, 40′, 60′) after the start of respective OLV with the randomised mode of ventilation (FCV or VCV) further measurements were performed (Fig. 1).

### Gas exchange, haemodynamics

Arterial and mixed venous blood samples were analysed for respiratory gases and pH using a blood gas analyser (ABL 80 Flex Basic, Radiometer, Denmark). Mean arterial and pulmonary artery pressures were measured continuously, whereas CO was determined with a pulmonary artery catheter by means of a conventional thermodilution method. Using the PICCO catheter extravascular lung water (EVLW) (lung function), intrathoracic blood volume (ITBV) and global end-diastolic blood volume (GEDV) (cardiac preload), systemic vascular resistance (SVR) (cardiac afterload) and stroke volume (SV) were determined. The intrapulmonary shunt fraction was calculated using a standard formula.

### Respiratory signals and regional pleural pressure

Airway flow was measured with the respective ventilator depending on the mode of ventilation. During VCV airway pressure was measured at the *y*-piece with a custom-made measurement system composed of a pressure transducer (163PC01D48-PCB, FirstSensors AG, Berlin, Germany) and respective hardware and software for amplification and recording (custom-build software written in LabVIEW, National Instruments, Austin, TX, USA). During FCV, the airway pressure signal was measured at the proximal tip of the Tritube and recorded by the Evone ventilator. The difference of location of airway pressure measurement between both ventilation modes was compensated before calculation of respiratory variables and respiratory mechanics: The pressure drop Δ*P* over the double-lumen tube was measured at different levels of airway flow during ventilation of an artificial lung. The determined flow (*F*) and Δ*P* values were fit to the Rohrer equation Δ*P*(*F*) = *K*_1_∙*F* + *K*_2_∙*F*^2^ [15, 16]. Δ*P*, as calculated from this equation during the experiments, was then subtracted from measured airway pressure during VCV.

Airway/tracheal pressure (*P*), airflow, and volume were recorded for 5 min at the end of each time point. Airway pressure and pleural pressure signals in ventral, dorsal, and caudal regions were obtained from pressure transducers [17] (163PC01D48-PCB, FirstSensors AG, Berlin, Germany). Respiratory system compliance and resistance were determined using multiple linear regression of the linear equation of motion composed of resistance *R* and compliance *C* two-compartmental model of the respiratory system. Regional transpulmonary pressures in the regions (ventral, dorsal, caudal) were calculated by subtracting the respective pleural pressure from airway pressure.

Mechanical work (MW) was determined through numerical integration of the inspiratory limb of the pressure-volume curve [18]. Mechanical power per cycle was derived by multiplying MW with the respiratory rate (RR): MP = MW∙RR [19]. MP is the sum of the dissipated energy in the airways over the resistance (resistive MP, MP_resis_) and the energy temporarily stored in the elastic lung tissue (elastic MP, MP_elast_) [18].

The corrected expired minute volume, a surrogate of ventilation efficiency, was calculated as the measured minute volume multiplied by the arterial partial pressure of carbon dioxide (PaCO_2_) divided by 40 mmHg [20].

### Electrical impedance tomography

Electrical impedance tomography (EIT) measurements were conducted with an operating frequency of 130 kHz and 50 frames s^−1^. Raw measured EIT data was 50 Hz filtered and reconstructed using the commercially available software of PulmoVista® 500 (Drägerwerk AG & Co. KGaA). Each EIT image of the resulting reconstructed temporal image series consisted of 32 × 32 pixels. EIT image reconstruction was done as described in detail by our group [21]. The global region of interest was a half-sphere covering the left hemisphere of the EIT and thus contained only the ventilated lung. This ROI was subdivided into three regions of interest (ROI) medial to lateral (independent, semi-dependent, and dependent). The difference between the end-inspiratory and end-expiratory image was taken to derive the tidal change of impedance. The ventro-dorsal centre of gravity of this tidal increase of impedance was therefore termed “centre of ventilation.”

### Statistical analyses

A sample size calculation was not carried out due to the exploratory nature of this substudy performed in the context of another yet unpublished experimental investigation on one-lung ventilation. Data are presented as mean and standard deviation if not stated otherwise. The statistical analysis was conducted with *R* Statistical programming language Version 3.4.1 [22]. Significance was accepted at *P* < 0.05. Differences between the two groups and the two ventilation modes were compared using linear mixed-effects [23], repeated measures ANOVA with with-in factors (ventilation mode and therapy time) and between factors (group and sequence of ventilation mode) [24].

## Results

### General aspects

Bodyweight (*P* = 0.805), total time of anaesthesia (*P* = 0.459), total time on mechanical ventilation (*P* = 0.544), cumulative doses of crystalloids (*P* = 0.892) and colloids (*P* = 0.154), total urine output (*P* = 0.264) and functional parameters at baseline did not differ significantly between normovolaemia and hypovolaemia animals. In the hypovolaemia group, 755 ± 80 mL blood was aspirated. The order of the sequence of the ventilation modes had no significant influence on any of the analysed variables.

### Gas exchange and haemodynamics

**Fig. 2:**
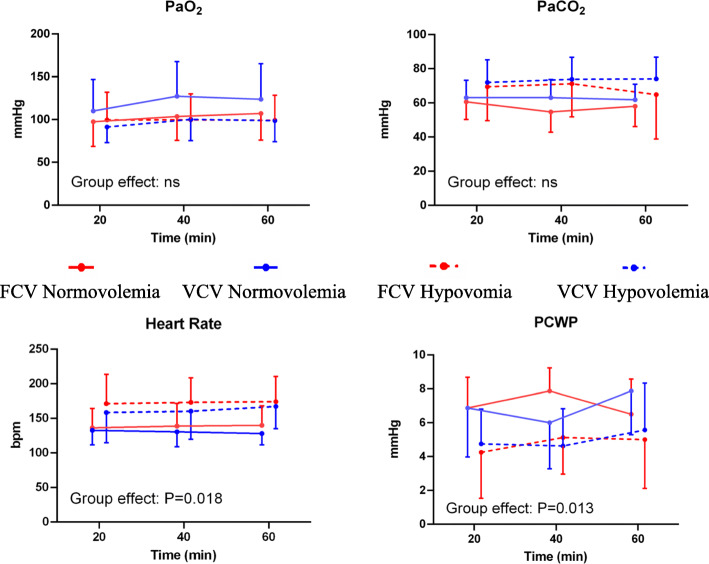
Gas exchange and haemodynamics. Symbols and vertical lines represent mean and standard deviation. Global statistical significance was accepted at *P* < 0.05. Differences between the two groups and the two ventilation strategies were compared using linear mixed-effects, repeated measures ANOVA. FCV = flow-controlled ventilation; VCV = volume-controlled ventilation; ns = not significant, PaO_2_ = arterial oxygen pressure; PaCO_2_ = arterial carbon dioxide pressure; PCWP = pulmonary capillary wedge pressure

PaO_2_ (*P* = 0.881), PaCO_2_ (*P* = 0.850), arterial pH (*P* = 0.652) and intrapulmonary shunt (*P* = 0.097) did not differ between volaemia groups and ventilation modes (Fig. 2, Table 1). Furthermore, MAP, MPAP, CO, EVLW, ITB, GEDV, and SVR did not differ between groups and ventilation modes (Fig. 2, Table 1). In the hypovolaemia group, heart rate (*P* = 0.018) and need for noradrenalin (*P* = 0.031) were higher and pulmonary capillary wedge pressure (*P* = 0.013) was lower compared with normovolaemia (Fig. 2).

**Table 1:** Gas exchange and haemodynamics

Parameter	Therapy				ANOVA (*P*=)				
Group	Mode	20′	40′	60′	Seq	Group	Mode	Time	Mixed
MAP (mmHg)					0.335	0.228	0.329	0.070	0.188
Norm	VCV	80 ± 13	76 ± 13	72 ± 14					
	FCV	74 ± 15	73 ± 17	78 ± 28					
Hypo	VCV	65 ± 20	65 ± 20	63 ± 22					
	FCV	69 ± 17	67 ± 17	64 ± 17					
MPAP (mmHg)					0.292	0.854	0.392	0.946	0.962
Norm	VCV	28 ± 8	26 ± 8	27 ± 7					
	FCV	27 ± 8	27 ± 6	27 ± 4					
Hypo	VCV	27 ± 7	28 ± 6	28 ± 5					
	FCV	26 ± 7	25 ± 6	25 ± 7					
CVP (mmHg)					0.380	0.066	0.368	0.738	0.875
Norm	VCV	4 ± 4	4 ± 4	5 ± 5					
	FCV	4 ± 3	3 ± 2	4 ± 2					
Hypo	VCV	2 ± 1	2 ± 2	2 ± 2					
	FCV	1 ± 2	2 ± 3	1 ± 2					
CO (l/min)					0.744	0.102	0.456	0.540	0.369
Norm	VCV	7.26 ± 1.95	7.19 ± 1.53	7.51 ± 1.21					
	FCV	7.22 ± 1.46	7.1 ± 1.42	6.82 ± 1.67					
Hypo	VCV	8.59 ± 3.74	8.68 ± 2.75	8.88 ± 2.57					
	FCV	8.95 ± 2.64	8.91 ± 2.37	8.76 ± 2.24					
EVLW (mL)					0.868	0.106	0.764	0.146	0.646
Norm	VCV	446 ± 69	455 ± 62	448 ± 61					
	FCV	438 ± 41	439 ± 41	438 ± 44					
Hypo	VCV	559 ± 179	536 ± 158	499 ± 127					
	FCV	562 ± 203	560 ± 169	530 ± 180					
ITBV (mL)					0.008	0.210	0.929	0.879	0.949
Norm	VCV	582 ± 167	566 ± 141	605 ± 173					
	FCV	587 ± 222	574 ± 240	560 ± 205					
Hypo	VCV	502 ± 90	484 ± 88	487 ± 96					
	FCV	501 ± 128	501 ± 118	540 ± 181					
GEDV (mL)					0.008	0.220	0.999	0.974	0.882
Norm	VCV	466 ± 134	450 ± 114	484 ± 138					
	FCV	470 ± 177	459 ± 192	449 ± 164					
Hypo	VCV	407 ± 70	387 ± 70	390 ± 77					
	FCV	401 ± 103	401 ± 94	432 ± 145					
SVR (dyn*s/cm^5^)					0.251	0.156	0.759	0.090	0.870
Norm	VCV	955 ± 294	878 ± 284	805 ± 236					
	FCV	1100 ± 436	858 ± 254	940 ± 242					
Hypo	VCV	736 ± 433	726 ± 410	682 ± 392					
	FCV	717 ± 306	720 ± 319	700 ± 325					
Noradrenalin (mg/h)					0.607	0.031	0.912	0.534	0.790
Norm	VCV	0.28 ± 0.50	0.78 ± 1.39	0.28 ± 0.50					
	FCV	0.23 ± 0.39	0.27 ± 0.48	0.28 ± 0.48					
Hypo	VCV	1.94 ± 2.19	2.04 ± 2.22	2.25 ± 2.59		0.054			
	FCV	2.12 ± 2.15	2.18 ± 2.28	2.19 ± 2.55		0.022			
Hb (mmol/L)					0.483	0.302	0.212	0.678	0.695
Norm	VCV	5.81 ± 0.91	5.64 ± 0.95	5.75 ± 1					
	FCV	5.92 ± 1.05	5.78 ± 1.07	5.69 ± 0.85					
Hypo	VCV	5.19 ± 0.97	5.11 ± 0.98	5.14 ± 0.91					
	FCV	5.46 ± 0.95	5.45 ± 0.94	5.44 ± 0.87					
Shunt (%)					0.944	0.103	0.097	0.050	0.114
Norm	VCV	32.8 ± 11.1	29.8 ± 8.9	29.9 ± 10.2					
	FCV	32.5 ± 8.2	32.2 ± 7.7	30.8 ± 8.2					
Hypo	VCV	41.7 ± 11.3	40.2 ± 11.6	38.3 ± 10.4					
	FCV	37 ± 9.8	37.7 ± 10.5	39.6 ± 12.1					

Values in mean ± standard deviation; Statistical *P* values according to linear mixed-effects repeated measures ANOVA with with-in subject factors: mode of ventilation (volume or flow-controlled ventilation) and time of therapy in minutes; and between subject factors group of therapy (normovolaemia or hypovolaemia) and randomisation sequence (volume or flow-controlled ventilation first). Significance was accepted for *P* < 0.05. Statistical analysis was performed in R statistical programming language and the package lme4

*FCV* flow-controlled ventilation, *VCV* volume-controlled ventilation, *MAP* mean arterial pressure, *MPAP* mean pulmonary arterial pressure, *CVP* central venous pressure, *CO* cardiac output, *EVLW* extravascular lung water, *ITBV* intrathoracic blood volume, *GEDV* global end-diastolic volume, *SVR* systemic vascular resistance, *Hb* haemoglobin, *Shunt* intrapulmonary shunt

### Respiratory system variables and mechanics

**Fig. 3:**
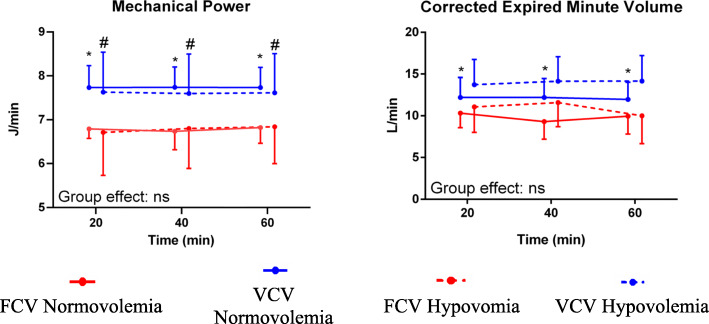
Mechanical power and corrected expired minute volume. Symbols and vertical lines represent mean and standard deviation. Global statistical significance was accepted at *P* < 0.05. Differences between the two groups and the two ventilation strategies were compared using linear mixed-effects, repeated measures ANOVA. FCV = flow-controlled ventilation; VCV = volume-controlled ventilation; ns = not significant

In the normovolaemia group, V_T_/kg body weight was higher during FCV (V_T_ 5.5 ± 0.4 ml/kg) than VCV (V_T_ 5.0 ± 0.3 ml/kg) (*P* = 0.042) (Table 2). The minute volume (*P* ≤ 0.001), RR (*P* ≤ 0.001), peak tracheal pressure (*P*_peak_) (*P* ≤ 0.001), total PEEP (PEEP + intrinsic PEEP) (*P* ≤ 0.001) (Table 2) and resistance of the respiratory system (*P* ≤ 0.001) were lower during FCV than VCV, irrespective of the volaemia status (Table 3). The compliance of the respiratory system was lower during FCV compared with VCV (*P* = 0.037) in the hypovolaemia group while it was not different in the normovolaemia group (*P* = 0.05) (Table 3). PEEP (*P* ≤ 0.001) (Table 2) and corrected expired minute volume (*P* = 0.022) (Fig. 3) were lower in the normovolaemia but not in the hypovolaemia group during FCV compared with VCV. In both volaemia groups, mechanical power (*P* ≤ 0.001) and resistive mechanical power (*P* ≤ 0.001) were lower during FCV than VCV, but elastic mechanical power was not different between the two modes (Fig. 3, Table 3). Regional pleural and transpulmonary pressures did not differ between groups or ventilation modes (Table 4). None of the respiratory parameters differed significantly between normovolaemia and hypovolaemia (Tables 2, 3 and 4).

**Table 2:** Respiratory parameters

Parameter	Therapy				ANOVA (*P*=)				
Group	Mode	20′	40′	60′	Seq	Group	Mode	Time	Mixed
V_T_ (mL/kg)					0.006	0.730	0.049	0.648	0.137
Norm	VCV	5.1 ± 0.2	5.1 ± 0.2	5.1 ± 0.2			0.042	0.978	0.044
	FCV	5.4 ± 0.3	5.6 ± 0.2	5.6 ± 0.2					
		*P* = 0.020	*P* < 0.001	*P* < 0.001					
Hypo	VCV	5.1 ± 0.3	5.1 ± 0.3	5.0 ± 0.3			0.251	0.621	0.532
	FCV	5.4 ± 0.2	5.5 ± 0.3	5.5 ± 0.4					
MV (L/min)					0.520	0.799	< 0.001	0.904	0.570
Norm	VCV	7.7 ± 0.5	7.7 ± 0.5	7.7 ± 0.5			< 0.001	0.894	0.995
	FCV	6.8 ± 0.2	6.8 ± 0.4	6.8 ± 0.4					
		*P* < 0.001	*P* < 0.001	*P* < 0.001					
Hypo	VCV	7.8 ± 0.7	7.8 ± 0.8	7.8 ± 0.8			< 0.001	0.778	0.453
	FCV	6.8 ± 1.0	7 ± 0.8	7.0 ± 0.8					
		*P* < 0.001	*P* = 0.008	*P* = 0.002					
RR (min^-1^)					0.897	0.495	< 0.001	0.989	0.403
Norm	VCV	35 ± 0	35 ± 0	35 ± 0			< 0.001	0.983	0.145
	FCV	29 ± 1	28 ± 1	29 ± 1					
		*P* < 0.001	*P* < 0.001	*P* < 0.001					
Hypo	VCV	35 ± 0	35 ± 0	35 ± 0			< 0.001	0.999	0.990
	FCV	28 ± 2	29 ± 2	28 ± 2					
		*P* < 0.001	*P* < 0.001	*P* < 0.001					
Pmean (cmH_2_O)					0.345	0.666	0.731	0.756	0.098
Norm	VCV	15.1 ± 1.1	14.8 ± 1.0	14.8 ± 1.1					
	FCV	15.3 ± 1.3	16 ± 1.4	15.9 ± 1.1					
Hypo	VCV	14.6 ± 1.8	14.8 ± 1.8	14.8 ± 1.8					
	FCV	14.9 ± 3.3	14.8 ± 3.2	15.6 ± 3.4					
Ppeak (cmH_2_O)					0.498	0.467	< 0.001	0.661	0.419
Norm	VCV	33 ± 3.1	32.3 ± 3.4	32.4 ± 3.3			< 0.001	0.590	0.082
	FCV	28 ± 3.4	29.6 ± 3.4	29.8 ± 3.4					
		*P* = 0.009	*P* = 0.133	*P* = 0.143					
Hypo	VCV	31.3 ± 3.4	31.8 ± 3.3	32.5 ± 4.3			0.027	0.315	0.715
	FCV	27.4 ± 5.8	26.5 ± 5.3	28 ± 4.8					
		*P* = 0.125	*P* = 0.033	*P* = 0.065					
PEEP (cmH_2_O)					0.265	0.141	0.002	0.977	0.634
Norm	VCV	5.1 ± 0.4	5.1 ± 0.4	5.1 ± 0.3			< 0.001	0.941	0.955
	FCV	4.2 ± 0.2	4.3 ± 0.1	4.2 ± 0.2					
		*P* < 0.001	*P* < 0.001	*P* < 0.001					
Hypo	VCV	4.9 ± 0.4	4.8 ± 0.3	4.9 ± 0.2			0.111	0.994	0.634
	FCV	3.7 ± 1.3	4 ± 1.2	3.5 ± 1.6					
Total PEEP (cmH_2_O)					0.386	0.947	< 0.001	0.885	0.420
Norm	VCV	6.2 ± 0.6	6.3 ± 0.7	6.3 ± 0.7			< 0.001	0.671	0.552
	FCV	4.43 ± 0.1	4.4 ± 0.1	4.3 ± 0.1					
		*P* < 0.001	*P* < 0.001	*P* < 0.001					
Hypo	VCV	6.6 ± 1.0	6.5 ± 0.7	6.5 ± 0.8			< 0.001	0.906	0.560
	FCV	4.2 ± 0.9	4.4 ± 0.9	4.0 ± 1.0					
		*P* < 0.001	*P* < 0.001	*P* < 0.001					

Values in mean ± standard deviation; Statistical *P* values according to linear mixed-effects repeated measures ANOVA with within subject factors: mode of ventilation (volume or flow-controlled ventilation) and time of therapy in minutes; and between subject factors group of therapy (normovolaemia or hypovolaemia) and randomisation sequence (volume or flow-controlled ventilation first). Significance was accepted for *P* < 0.05. Statistical analysis was performed in R Statistical programming language and the package lme4

*FCV* flow-controlled ventilation, *VCV* volume-controlled ventilation, *V*_*T*_ tidal volume, *MV* minute volume, *RR* respiratory rate, *P*_*mean*_ mean tracheal pressure, *P*_*peak*_ peak tracheal pressure, *PEEP* positive end-expiratory pressure, *total PEEP* PEEP + intrinsic PEEP

**Table 3:** Respiratory mechanics and mechanical power

Parameter	Therapy				ANOVA (*P*=)				
Group	Mode	20′	40′	60′	Seq	Group	Mode	Time	Mixed
R (cmH_2_O s/L)					0.171	0.531	< 0.001	1.000	0.896
Norm	VCV	32.7 ± 7.59	33.3 ± 7.81	32.8 ± 7.53			< 0.001	0.977	0.963
	FCV	7.26 ± 0.973	8.1 ± 3.29	7.47 ± 1.73					
		*P* < 0.001	*P* < 0.001	*P* < 0.001					
Hypo	VCV	35 ± 9.18	34.5 ± 11	34.9 ± 11.1			< 0.001	0.986	0.860
	FCV	8.58 ± 8.49	8.08 ± 6.78	7.44 ± 6.26					
		*P* = 0.003	*P* = 0.004	*P* = 0.003					
C (mL/cmH_2_O)					0.882	0.142	0.006	0.980	0.860
Norm	VCV	11 ± 1.77	11.7 ± 2.06	11.6 ± 2.19			0.050	0.347	0.849
	FCV	9.34 ± 1.61	9.7 ± 1.23	9.72 ± 1.57					
Hypo	VCV	12.9 ± 2.36	12.3 ± 2.13	12.3 ± 2.29			0.037	0.175	0.969
	FCV	11.5 ± 2.13	11 ± 1.8	10.9 ± 1.63					
		*P* = 0.042	*P* = 0.013	*P* = 0.041					
MP_resis_ (J/min)					0.375	0.521	< 0.001	0.840	0.901
Norm	VCV	6.5 ± 1.3	6.6 ± 1.4	6.4 ± 1.4			< 0.001	0.855	0.787
	FCV	1.2 ± 0.2	1.3 ± 0.4	1.3 ± 0.3					
		*P* < 0.001	*P* < 0.001	*P* < 0.001					
Hypo	VCV	7.2 ± 2.1	7.0 ± 2.3	7.1 ± 2.3			< 0.001	0.899	0.992
	FCV	1.3 ± 0.9	1.2 ± 0.7	1.2 ± 0.7					
		*P* < 0.001	*P* = 0.001	*P* < 0.001					
MP_elas_ (J/min)					0.392	0.437	0.243	0.931	0.206
Norm	VCV	7.6 ± 1.2	7.4 ± 1.4	7.4 ± 1.4					
	FCV	8.4 ± 1.4	8.7 ± 1.6	8.9 ± 1.7					
Hypo	VCV	6.9 ± 1.8	7.1 ± 1.5	7.1 ± 1.5					
	FCV	7.5 ± 2.9	8.0 ± 2.4	8.1 ± 2.5					

Values in mean ± standard deviation; Statistical *P* values according to linear mixed-effects repeated-measures ANOVA with within subject factors: mode of ventilation (volume or flow-controlled ventilation) and time of therapy in minutes; and between subject factors group of therapy (normovolaemia or hypovolaemia) and randomisation sequence (volume or flow-controlled ventilation first). Significance was accepted for *P* < 0.05. Statistical analysis was performed in R statistical programming language and the package lme4.

*FCV* flow-controlled ventilation, *VCV* volume-controlled ventilation, *C* compliance of the respiratory system, *MP*_*resis*_ resistive mechanical power, *MP*_*elas*_ elastic mechanical power

**Table 4:** Regional end-expiratory pleural and transpulmonary pressures

Parameter	Therapy				ANOVA (*P*=)				
Group	Mode	20′	40′	60′	Seq	Group	Mode	Time	Mixed
PPvent (cmH_2_O)					0.073	0.994	0.022	0.437	0.116
Norm	VCV	0.1 ± 2.6	− 0.1 ± 2.7	− 0.0 ± 3.2			0.076	0.866	0.256
	FCV	− 1.3 ± 3.6	− 1.0 ± 3.4	− 0.2 ± 3.5					
Hypo	VCV	0.3 ± 0.7	− 0.6 ± 2.4	− 0.3 ± 2.9			0.161	0.327	0.288
	FCV	− 0.7 ± 2.1	− 0.9 ± 3.0	− 0.3 ± 1.7					
PPdors (cmH_2_O)					0.054	0.079	0.975	0.045	0.744
Norm	VCV	− 1.9 ± 3.1	− 1.5 ± 4.1	0.1 ± 3.0					
	FCV	− 1.0 ± 3.1	− 1.6 ± 3.7	− 0.8 ± 2.9					
Hypo	VCV	0.9 ± 0.7	0.6 ± 1.5	1.1 ± 0.8					
	FCV	− 0.3 ± 3.2	0.6 ± 0.9	1.1 ± 0.8					
PPcaud (cmH_2_O)					0.754	0.109	0.211	0.202	0.447
Norm	VCV	− 0.2 ± 3.2	− 0.7 ± 3.5	0.1 ± 3.3					
	FCV	− 1.8 ± 4	− 0.8 ± 3.4	− 0.0 ± 3.4					
Hypo	VCV	1.0 ± 2.0	1.9 ± 1.4	1.7 ± 1.3					
	FCV	1.3 ± 1.6	1.5 ± 1.4	1.5 ± 1.6					
TPvent (cmH_2_O)					0.103	0.970	0.262	0.461	0.076
Norm	VCV	5.4 ± 2.5	5.6 ± 2.8	5.4 ± 3.1					
	FCV	5.8 ± 3.5	5.3 ± 3.2	4.5 ± 3.2					
Hypo	VCV	5.11 ± 0.7	6 ± 2.4	5.7 ± 2.8					
	FCV	5.22 ± 2.2	5.4 ± 3.0	4.8 ± 1.6					
TPdors (cmH_2_O)					0.092	0.098	0.347	0.036	0.984
Norm	VCV	7.3 ± 3.0	7.0 ± 4.4	5.3 ± 2.8					
	FCV	5.8 ± 3.5	5.5 ± 3.2	5 ± 2.7					
Hypo	VCV	4.5 ± 0.6	4.8 ± 1.6	4.3 ± 0.8					
	FCV	4.9 ± 2.8	3.9 ± 0.8	3.36 ± 1.3					
TPcaud (cmH_2_O)					0.626	0.060	0.839	0.311	0.953
Norm	VCV	5.8 ± 3.0	6.1 ± 3.6	5.3 ± 3.2					
	FCV	6.6 ± 4.4	6.0 ± 3.9	5.6 ± 4.4					
Hypo	VCV	4.4 ± 1.9	3.5 ± 1.3	3.7 ± 1.2					
	FCV	3.2 ± 1.8	3.0 ± 1.6	3.0 ± 2.0					

Values in mean ± standard deviation; Statistical *P* values according to linear mixed-effects repeated measures ANOVA with within subject factors: mode of ventilation (volume or flow-controlled ventilation) and time of therapy in minutes; and between subject factors group of therapy (normovolaemia or hypovolaemia) and randomization sequence (volume or flow-controlled ventilation first). Significance was accepted for *P* < 0.05. Statistical analysis was performed in R statistical programming language and the package lme4

*FCV* flow-controlled ventilation, *VCV* volume-controlled ventilation, *PIPvent* end-expiratory ventral pleural pressure, *PIPdors* end-expiratory dorsal pleural pressure, *PIPcaud* end-expiratory caudal pleural pressure, *TPvent* end-expiratory ventral transpulmonary pressure, *TPdors* end-expiratory dorsal transpulmonary pressure, *TPcaud* end-expiratory caudal transpulmonary pressure

### Regional ventilation

Regional ventilation of the left lung did not differ significantly between FCV and VCV during both normovolaemia and hypovolaemia (Table 5).

**Table 5:** Distribution of tidal impedance changes and centre of ventilation in the therapy lung

Parameter	Mode	Therapy			ANOVA (*P*=)				
Group		20′	40′	60′	Seq	Group	Mode	Time	Mixed
Indepen (%)					0.791	0.596	0.238	0.291	0.791
Normo	VCV	41.4 ± 8.71	41.5 ± 8.45	41.9 ± 8.47					
	FCV	41.1 ± 10.5	42.7 ± 10.8	44 ± 10.4					
Hypo	VCV	39.1 ± 11	39.4 ± 10.9	38.4 ± 11.3					
	FCV	39.1 ± 11.9	39.7 ± 12.2	40.1 ± 12.8					
Semidep (%)					0.922	0.616	0.145	0.437	0.127
Normo	VCV	56.3 ± 7.61	56.2 ± 7.38	55.7 ± 7.34					
	FCV	56.4 ± 9.59	54.9 ± 9.91	53.6 ± 9.62					
Hypo	VCV	58.1 ± 10.2	57.8 ± 10.2	58.4 ± 10.2					
	FCV	58.1 ± 11.2	57.6 ± 11.6	57.3 ± 12.3					
Depend (%)					0.962	0.408	0.698	0.731	0.561
Normo	VCV	2.28 ± 1.25	2.29 ± 1.23	2.31 ± 1.33					
	FCV	2.53 ± 1.07	2.4 ± 0.974	2.4 ± 0.927					
Hypo	VCV	2.83 ± 1.69	2.83 ± 1.76	2.57 ± 1.75					
	FCV	2.8 ± 1.56	2.63 ± 1.55	2.58 ± 1.55					
Centre of ventilation (%)					0.208	0.585	0.103	0.090	0.208
Normo	VCV	60.5 ± 3.88	60.5 ± 3.77	61.2 ± 3.16					
	FCV	60.8 ± 3.51	62.2 ± 2.63	62.3 ± 3.09					
Hypo	VCV	60.2 ± 4.28	60.4 ± 4.46	60.3 ± 4.68					
	FCV	60.3 ± 5.02	60 ± 4.03	60.4 ± 3.92					

Values in mean ± standard deviation; Statistical *P* values according to linear mixed-effects repeated measures ANOVA with within subject factors: mode of ventilation (volume or flow-controlled ventilation) and time of therapy in minutes; and between subject factors group of therapy (normovolaemia or hypovolaemia) and randomization sequence (volume or flow-controlled ventilation first). Significance was accepted for *P* < 0.05. Statistical analysis was performed in R statistical programming language and the package lme4

*FCV* flow-controlled ventilation, *VCV* volume-controlled ventilation, *Indep* independent lung regions, *semidep* semidependent lung regions, *dpend* dependent lung regions. Centre of ventilation was calculated with the base at dorsal part of the lung. 0% would mean ventilation (change of impedance due to ventilation) is concentrated on the most dependent layer

## Discussion

The main findings of the present study were that, in a model of thoracic surgery with normovolaemia as well as hypovolaemia in pigs under OLV, FCV, as compared to VCV, reduced the mechanical power mainly due to the resistive mechanical power component without significant difference in oxygenation. Additionally, with FCV higher ventilation efficiency was achieved during normovolaemia.

To our knowledge, this is the first study reporting the use of FCV during OLV in a clinically relevant model of thoracic surgery during intravascular normo- and hypovolaemia. An important strength of this study is that we were able to reproduce key features of surgical thoracic procedures including systemic inflammation. At the dose used in this study, LPS results in a surgery-like inflammatory response, while not altering haemodynamics importantly [25]. Another strength of our study is that the investigation included scenarios of normovolaemia and moderate to severe hypovolaemia [26], both of which may occur in clinical practice [27], with different effects on hypoxic pulmonary vasoconstriction and intrapulmonary shunt [13].

Our observation that FCV did not improve oxygenation contrasts with a previous report. In pigs without lung injury, FCV increased oxygenation compared with VCV during two-lung ventilation [10]. A possible explanation for this discrepancy is that the mean tracheal pressure (*P*_mean_) was higher during FCV in that study [10], whereas *P*_mean_ did not differ significantly between FCV and VCV in the present trial. Since higher mean tracheal pressures resulted in more open lung units [28], the intrapulmonary shunt was reduced and PaO_2_ increased. Another possible reason is that VCV resulted in higher total PEEP during VCV than FCV in our animals. Intrinsic PEEP can be more effectively avoided during FCV due to the active control of the expiration by the ventilator, while a passive expiratory flow combined with a relatively narrow inner diameter of the double-lumen tube during VCV led to an unintended increase of intrinsic PEEP [29]. The higher intrinsic PEEP during VCV may have led to air trapping, which can improve oxygenation, but not necessarily ventilation. In fact, the EIT analysis showed that the distribution of ventilation did not differ significantly between VCV and FC, which is in line with the regional distribution of end-expiratory pleural and transpulmonary pressures that did not differ significantly between VCV and FCV. In contrast to the present study, in pigs with non-injured and injured lungs, FCV led to a shift of regional ventilation towards dependent lung areas [11]. However, in that study, *P*_mean_ was also higher during FCV than VCV [11].

Our finding that PaCO_2_ did not differ between FCV and VCV is in line with a previous study in pigs with non-injured lungs [10]. Nevertheless, in our study, the minute volume was markedly lower during FCV than VCV, yielding a higher ventilation efficiency under normovolaemia, and suggesting a lower alveolar dead-space ventilation during FCV. This is supported by the fact that the pulmonary shunt was not different between groups. The increased ventilation efficiency of FCV might be explained by improved emptying of lung units with different time constants at lower expiratory flow. Moreover, expiratory alveolar pressure is kept for a longer time above the alveolar closing pressure at the lower constant expiratory flow. However, we cannot completely rule out the possibility that a reduction in the anatomic dead space due to the relatively small inner diameter of the Tritube, as compared to the inner diameter of the double-lumen tube, also contributed to this effect. During hypovolaemia, ventilation efficiency was not different between FCV and VCV. This might be explained by the fact that alveolar dead space is increased due to pulmonary hypoperfusion secondary to hypovolaemia [30] blunting possible positive effects of expiratory flow control.

In the present study, the haemodynamic impact of FCV and VCV did not differ significantly, irrespective of the volaemia status. Contrastingly in pigs with hypovolaemia induced by haemorrhage, FCV improved venous return and cardiac output compared to VCV [31]. However, differently from the present investigation, the end-expiratory pressure during FCV was negative in that study, while our animals were ventilated with a PEEP of 5 cmH_2_O in both ventilation modes.

Our discovery that the compliance of the respiratory system was similar for FCV and VCV during normovolaemia is in line with other experimental studies [10, 11]. However, in the hypovolaemia group, compliance was lower for FCV as compared with VCV. This might be explained by a higher total PEEP during VCV, which can result in a shift on the pressure-volume curve to a more compliant region of the lung during VCV.

Our finding that FCV reduced the resistance of the respiratory system compared with VCV is in line with previous reports [12, 28]. In this manner, FCV also reduced mechanical power and resistive mechanical power compared to VCV. This reduction is particularly important since during OLV resistance is increased already due to decreased lung volume, the lateral decubitus position and the displacement of the diaphragm and the mediastinum and further rises depending on the gas flow. High-resistive mechanical power due to high gas flow, as found during VCV, can be associated with shear stress at the top of the cells within the respiratory bronchi [32] and therefore with increased risk for lung damage [5, 33]. Resistive mechanical power relates to the inspiratory phase only. However, the energy accumulated at end-inspiration will be dissipated both into the lung structures and into the atmosphere at end-expiration. Since inspiration and expiration showed similar constant low flow profiles during FCV, the amount of resistive energy dissipated in the lungs during a ventilation cycle was most likely further minimised. In fact, in a porcine acute lung injury model flow-controlled expiration resulted in reduced ventilator-induced lung injury [34].

### Potential implications of these findings

OLV is associated with an increased risk of hypoxemia and VILI formation. A mode of ventilation that avoids both is urgently needed. The present findings suggest that FCV in OLV maintains gas exchange while reducing mechanical stress on the lungs, possibly reducing postoperative pulmonary complications. Further studies are necessary to clarify whether these physiological benefits also lead to a better clinical outcome.

### Limitations

The present study knows several limitations. First, the used thoracic surgery model does not fully represent the clinical scenario, especially due to the lack of surgical manipulation of the lungs and its potential effects on atelectasis in the dependent lung. Thus, we cannot extrapolate our findings directly to patients. Second, we addressed only short-term effects of FCV and VCV during OLV. Nevertheless, one-lung ventilation is usually limited to short time periods. Third, we did not assess gas exchange variables at 30 minutes after initiation of OLV, when the hypoxic pulmonary vasoconstriction reaches a maximum [13]. However, if an effect in gas exchange was overseen, the importance of such effect is questionable. Fourth, the crossover design of our study precluded the comparison of effects of ventilation modes on VILI. Thus, we do not know whether reduced mechanical power would translate into less lung damage.

## Conclusions

In a model of OLV in normo- and hypovolemic pigs, mechanical power was lower during FCV compared to VCV, without significant differences in oxygenation. Furthermore, the efficacy of ventilation was higher during FCV compared to VCV during normovolaemia.

## Data Availability

The datasets used and/or analysed during the current study are available from the corresponding author on reasonable request.

## Abbreviations

C: Compliance of the respiratory system
CO: Cardiac output
EIT: Electrical impedance tomography
EVLW: Extravascular lung water
FCV: Flow-controlled ventilation
FIO2: Inspired fraction of oxygen
GEDV: Global end-diastolic volume
I:E: Inspiratory to expiratory time ratio
IPPV: Intermittent positive pressure ventilation
ITBV: Intrathoracic blood volume
LPS: Lipopolysaccharide
MP: Mechanical power
MPresis: Resistive mechanical power
MPelas: Elastic mechanical power
MV: Minute volume
OLV: One-lung ventilation
PEEP: Positive end-expiratory pressure
PIP: Peak inspiratory pressure
Pmean: Mean tracheal pressure
PP: Pleural pressure
Ppeak: Peak tracheal pressure
R: Resistance of the respiratory system
RR: Respiratory rate
RR: Respiratory rate
SVR: Systemic vascular resistance
TLV: Two-lung ventilation
TP: Transpulmonary pressure
VCV: Volume-controlled ventilation
VILI: Ventilator-induced lung injury
VT: Tidal volume
